# Knowledge and Practices of Toxoplasmosis among Clinical Laboratory Professionals: A Cross-Sectional Study in Durango, Mexico

**DOI:** 10.3390/ijerph14111413

**Published:** 2017-11-18

**Authors:** Cosme Alvarado-Esquivel, Luis Francisco Sánchez-Anguiano, Luis Omar Berumen-Segovia, Jesús Hernández-Tinoco, Yazmin del Rosario Rico-Almochantaf, Alfredo Cisneros-Camacho, Jorge Arturo Cisneros-Martínez

**Affiliations:** 1 Faculty of Medicine and Nutrition, Juárez University of Durango State, Avenida Universidad S/N, 34000 Durango, Mexico; drluisomarberumen@gmail.com (L.O.B.-S.); yazminr347@gmail.com (Y.d.R.R.-A.); alfredo2oper@hotmail.com (A.C.-C.); jorgecisner10@yahoo.com.mx (J.A.C.-M.); 2 Institute for Scientific Research “Dr. Roberto Rivera Damm”, Juárez University of Durango State, Avenida Universidad S/N, 34000 Durango, Mexico; lfsanguiano@hotmail.com (L.F.S.-A.); jhtinoco@yahoo.com (J.H.-T.)

**Keywords:** toxoplasmosis, knowledge, practices, laboratory professionals, Mexico

## Abstract

*Background*: The aim of this study was to determine the level of knowledge and practices about toxoplasmosis in a sample of clinical laboratory professionals in Mexico. *Methods*: 192 clinical laboratory professionals were surveyed. They were asked about (1) *Toxoplasma gondii*; (2) clinical manifestations, diagnosis, treatment, and epidemiology of toxoplasmosis; and (3) their practices with respect to toxoplasmosis. *Results*: The range of animals infected by *T. gondii* was known by 44.8% of participants. Clinical aspects of toxoplasmosis were known by up to 44.3% of subjects. Correct answers about the interpretation of serological markers of *T. gondii* infection were provided by up to 32.8% of participants. A minority (32.2%) of participants knew about a high number of false positive results of anti-*T. gondii* IgM antibody tests. Most participants (90.1%) did not know what the anti-*T. gondii* IgG avidity test was. Up to 55.7% of participants provided incorrect answers about the interpretation of serology tests for the treatment of pregnant women. Common routes of *T. gondii* infection were known by <15% of participants. Most (84.4%) participants had not performed tests for detection *T. gondii* infection. *Conclusions*: Results indicate incomplete knowledge of *T. gondii* infection and toxoplasmosis and a limited practice of laboratory tests among the professionals surveyed.

## 1. Introduction

The parasite *Toxoplasma gondii* (*T. gondii*) causes infections in humans and animals, and is highly prevalent throughout the world [1,2,3]. Nearly one-third of the human population has been infected with *T. gondii* [4]. Transmission of *T. gondii* occurs mainly by ingestion of meat containing tissue cysts or by consumption of water or food containing oocysts shed by cats [5]. The definitive host of *T. gondii* are felids [6], and this intracellular parasite infects a wide range of warm-blooded intermediate hosts [7]. Other routes of *T. gondii* infections include congenital [8], blood transfusion [9], or organ transplant [10]. Most infections with *T. gondii* are asymptomatic [11]. However, toxoplasmosis, the disease caused by *T. gondii*, is characterized by retinochoroiditis [12] or cervical lymphadenopathy [5]. In immunocompromised individuals, a reactivation of a latent *T. gondii* infection may lead to life-threatening encephalitis [5]. Primary *T. gondii* infection in pregnant women can cause miscarriage [13] or fetal damage [14]. Routine tests for the diagnosis of *T. gondii* infection are based on serology [15]. Enzyme-linked immunosorbent assays and enzyme-linked fluorescent assays are tests currently used for detection of anti-*T. gondii* IgG and IgM antibodies in humans [16]. *T. gondii* commercial IgM diagnostic test kits can yield a number of false-positive results [17,18]. Anti-*T. gondii* IgM antibodies can persist for several years and a chronic *T. gondii* infection can be erroneously classified as an acute infection if diagnosis is based on IgM serology only [18]. Avidity tests of specific anti-*T. gondii* IgG antibodies are an important tool for discrimination of recent and past infections, especially in pregnant women [19,20]. Other serological tests for the diagnosis of *T. gondii* infection, i.e., Western blot [21] and indirect immunofluorescence test [22], are used less frequently. Confirmation of the presence of anti-*T. gondii* antibodies has been performed by Western blot [23]. Polymerase-chain-reaction-based molecular techniques are also useful for the diagnosis of *T. gondii* infection [24]. 

There is currently no report about knowledge and practices of toxoplasmosis among laboratory workers. Physicians, chemists, biologists, and technicians are professionals usually working in clinical laboratories, and it is important to know the knowledge and practices about toxoplasmosis because this would help to diagnose *T. gondii* infection by performing specific *T. gondii* laboratory tests. This information is useful for the design of optimal strategies for education and health care. The aim of the present study was to determine the level of knowledge and practices of toxoplasmosis in a sample of laboratory professionals in Mexico. 

## 2. Materials and Methods

### 2.1. Study Design and Population Studied

We performed a cross-sectional study of laboratory professionals attending the XVI National Congress of Clinical Chemistry and Laboratory Medicine held in 2016 in Durango City, Mexico. Inclusion criteria for enrollment were as follows: (1) physicians, chemists, biologists, and technicians working in clinical laboratories in Mexico; (2) those who voluntarily participated in the survey. Age, gender, type of area in which the professionals work (urban, suburban, or rural), or whether they work for private and/or public organizations were not restrictive criteria for enrollment. In total, 192 participants were surveyed. General characteristics of the study population are shown in Table 1.

### 2.2. Questionnaire

A questionnaire was designed to record general data of participants, as well as their knowledge and practices of toxoplasmosis. The questionnaire was anonymous and self-administered. Most questions of the questionnaire were multiple-choice questions, and some were open-ended questions. Items of general data are shown in Table 1. Items to assess the knowledge and practices of toxoplasmosis included questions about the infectious agent, clinical aspects of the infection (Table 2), questions about the laboratory diagnosis and treatment (Table 3), questions about the epidemiology of *T. gondii* infection (Table 4), and questions about the practices with respect to toxoplasmosis at their workplace (Table 5). 

### 2.3. Statistical Analysis

The statistical analysis was performed with the aid of the software Epi Info 7 (Centers for Disease Control and Prevention, Atlanta, GA, USA), and SPSS 15.0 (SPSS Inc. Chicago, IL, USA). Descriptive statistics (percentages) were used to summarize and describe the data. Correlation of variables was analyzed by the Pearson’s chi-square test and Fisher´s exact test (when values were small). A *p*-value < 0.05 was considered statistically significant.

## 3. Results

### 3.1. General Data of Participants

Most participants were female (73.4%), worked in urban areas (93.8%), worked in public laboratories (93.8%), worked in Durango State (56.8%), were chemists or biologists (60.4%), were younger than 30 years old, and had less than 10 years of seniority in terms of their work (58.9%) (Table 1). 

#### 3.1.1. Knowledge of the Parasite and Clinical Aspects of Toxoplasmosis

Results about the knowledge and clinical characteristics of toxoplasmosis are shown in Table 2. Most participants answered correctly that *T. gondii* is a parasite (89.6%), widely distributed in the world (76.6%), and having felids as definitive hosts (83.8%). In contrast, less than half of the participants knew that *T. gondii* infects a wide range of animals and birds (44.8%). Encephalitis was the most (44.3%) frequently recognized clinical characteristic of toxoplasmosis. 

#### 3.1.2. Knowledge of the Diagnosis and Treatment of Toxoplasmosis

Results about the knowledge of diagnosis and treatment of toxoplasmosis are shown in Table 3. Concerning interpretation of *T. gondii* serology, 32.8% of participants answered correctly that a positive test for IgG with a negative test for IgM means a chronic infection. The question that the routine test for detection of anti-*T. gondii* IgM antibodies in serum yields a high number of false positive results was answered correctly by 32.2% of participants. The great majority of participants (90.1%) did not know what an anti-*T. gondii* IgG avidity tests was, and 90.6% did not know what an avidity test was used for. Knowledge of avidity tests was similar among participants regardless of their type of training (*p* = 0.83) or the amount of seniority they had (*p* = 0.29). No other tests for the diagnosis of *T. gondii* infection were known by 83.9% of participants. Of those who knew about other tests for the diagnosis of *T. gondii* infection, 45.2% provided a good answer about *T. gondii* tests. Most (55.7%) participants answered that the demonstration of anti-*Toxoplasma* IgG and IgM antibodies in the serum of a pregnant woman was enough reason to provide treatment, and 42.2% of participants answered that the demonstration of anti-*Toxoplasma* IgM antibodies without IgG in the serum of a pregnant woman was enough reason to provide treatment. 

#### 3.1.3. Knowledge of the Epidemiology of Toxoplasmosis

Results about the knowledge of the epidemiology of toxoplasmosis are shown in Table 4. Half (50%) of the participants answered that contact with cats was a route of *T. gondii* infection, whereas ingestion of water, raw or undercooked meat, and unwashed fruits or vegetables were choices answered by less than 15% of participants. With respect to the world prevalence of *T. gondii* infection, 19.8% of participants answered that up to one-third of the population was infected by *T. gondii*.

#### 3.1.4. Practices about Toxoplasmosis

Results about the practices of toxoplasmosis are shown in Table 5. The great majority (84.4%) of participants had not performed tests for detection of infection with *T. gondii* at their workplace. Concerning the question of which tests were used in their laboratory for detection of infection with *T. gondii* in pregnant women, 57.3% answered that anti-*T. gondii* IgG and IgM tests. With respect to the performance of confirmatory tests for *T. gondii* infection, 92.7% answered no performance. However, of 14 participants who answered that confirmatory tests were performed in their workplace, only 4 (28.6%) provided a good answer about the type of confirmatory tests used. Most (91.7%) participants answered that no molecular tests for *T. gondii* infection were performed in their workplace. Of 16 participants who answered that molecular tests were performed in their workplace, 9 (56.3%) provided a good answer about the type of molecular tests used.

## 4. Discussion

Studies about the knowledge and practices of toxoplasmosis among health care professionals are scanty. We are not aware of any study about the knowledge and practices of toxoplasmosis among laboratory professionals. Therefore, this study was aimed to determine the knowledge and practices about toxoplasmosis among a sample of laboratory professionals attending a national congress of clinical chemistry and laboratory medicine in Durango City, Mexico. Results suggest that most laboratory professionals had good knowledge of the general characteristics of *T. gondii* since 89.6% of participants knew that *T. gondii* is a parasite, 76.6% knew that it is distributed worldwide, and 83.8% knew that felids are the definitive hosts of *T. gondii*. However, less than half (44.8%) of participants knew that *T. gondii* infects a wide range of animals and birds. With respect to clinical characteristics of toxoplasmosis, only one question, including well recognized clinical features of toxoplasmosis, was asked. Less than 30% of participants knew that lymphadenopathy and ocular disease occurs in toxoplasmosis, whereas 44.3% of participants knew that encephalitis occurs in toxoplasmosis. However, encephalitis occurs mostly in immunocompromised patients [5] and therefore, it is not a common clinical manifestation of toxoplasmosis. Results thus suggest a suboptimal knowledge of basic clinical characteristics of toxoplasmosis among our study population. Concerning questions of the knowledge of the interpretation of laboratory results for the diagnosis and treatment of toxoplasmosis, a limited number of participants answered correctly. The only correct answer about the interpretation of laboratory tests, which was that a positive test for IgG with a negative test for IgM anti-*T. gondii* indicates a chronic infection, was given by 32.8% of participants. The statement that the routine test for detection of anti-*Toxoplasma* IgM antibodies in serum yields a high number of false positive results was correctly chosen by 32.3% of participants. Remarkably, only 9.9% of participants knew the anti-*T. gondii* IgG avidity test, and only 2.6% of those who knew it had knowledge of what it was used for. The vast majority (83.9%) did not know about tests other than the IgG and IgM tests for the diagnosis of *T. gondii* infection. With respect to questions about the interpretation of laboratory tests to justify the treatment, only 18.8% of participants correctly answered that it was false that the demonstration of anti-*T. gondii* IgG and IgM antibodies in the serum of a pregnant woman is enough reason to provide treatment. Anti-*T. gondii* IgM antibodies can be present for years and do not necessarily indicate acute infection in pregnant women [18]. Similarly, only 29.2% of participants knew that the demonstration of anti-*T. gondii* IgM antibodies without IgG in the serum of a pregnant woman is not enough reason to provide treatment. All pregnant women with a positive anti-*T. gondii* IgM test should be further examined with the anti-*T. gondii* IgG avidity test to discriminate between acute and past infections [19,20]. Only women with low anti-*T. gondii* IgG avidity should be considered for treatment [19,20]. Poor knowledge of the avidity test found in the present study has also been reported with respect to physicians attending pregnant women in Mexico [25] and the USA [26]. 

With respect to knowledge of the epidemiology of toxoplasmosis, many participants did not correctly answer questions about important routes of infection with *T. gondii*. *T. gondii* infection can be acquired by ingestion of water or food contaminated with oocysts shed by cats and by the consumption of raw or undercooked meat containing tissue cysts [5]. In fact, *T. gondii* infection has been associated with consumption of unwashed raw vegetables [27], fruit [28], and untreated water [29] in Mexico. The most frequent answer regarding the transmission of *T. gondii* given by the participants was contact with cats. The factor presence of cats at home has been associated with *T. gondii* infection in Durango, Mexico [29,30,31,32]. However, direct contact with cats is not thought to be a primary risk for human infection [33]. It is estimated that nearly one-third of the world’s population is infected with *T. gondii* [4]. In this study, only 19.8% of participants answered correctly that up to one-third of the population is infected with *T. gondii*. The results thus indicate that the laboratory professionals surveyed had incomplete knowledge of the epidemiology of *T. gondii* infection. 

With respect to the practices of toxoplasmosis, most participants stated that no laboratory tests for detection of *T. gondii* infection are performed in their workplace. This finding may reflect the scanty use of tests for the diagnosis of *T. gondii* infection in clinical laboratories in Mexico. The screening of *T. gondii* exposure is based on the determination of anti-*T. gondii* IgG and IgM antibodies [15,16]. However, in the present study, only 57.3% of participants informed that these infection markers were performed to detect *T. gondii* infection in pregnant women in their laboratories. The use of serology tests for the confirmation of *T. gondii* infection or molecular tests to detect *T. gondii* DNA is very rare in Mexico as reported by the great majority of participants surveyed. Results thus indicate that laboratory professionals surveyed had a limited practice about clinical laboratory tests for the diagnosis of *T. gondii* infection. 

Our study has some limitations. We studied only attendees of a national congress and most of them were working in one state (Durango) of Mexico. These results thus cannot be extrapolated to laboratory professionals in the whole country. 

## 5. Conclusions

This is the first study on knowledge and practices of toxoplasmosis among laboratory professionals. Results indicate incomplete knowledge of *T. gondii* infection and toxoplasmosis including interpretation of laboratory tests, clinical features, epidemiology and treatment, and a limited practice of laboratory tests among the professionals surveyed. These results can be useful for the design of strategies leading to optimal education about clinical laboratory tests for the diagnosis of *T. gondii* infection. 

## Figures and Tables

**Table 1:** General characteristics of the population studied.

Characteristic	No.	%
Workplace		
Durango	109	56.8
Other Mexican State	83	43.2
Type of training		
Chemist or biologist	116	60.4
Laboratory technician	63	32.8
Physician	3	1.6
Other *	10	5.2
Age (years old)		
<0	89	46.4
30–50	67	34.9
>50	32	16.6
Unknown	4	2.1
Gender		
Female	141	73.4
Male	51	26.6
Type of work area		
Urban	180	93.8
Suburban	10	5.2
Rural	2	1.0
Type of professional work		
Public only	180	93.8
Private only	10	5.2
Public and private	2	1
Amount of seniority (years)		
<10	113	58.9
10–20	43	22.4
>0	36	18.8

* Student, Professor.

**Table 2:** Knowledge of *Toxoplasma gondii* and clinical aspects of toxoplasmosis.

Questions and Answers	No.	%
*Toxoplasma gondii* is:		
A virus	6	3.1
A bacterium	6	3.1
A parasite	172	89.6
A fungus	4	2.1
I do not know.	4	2.1
*Toxoplasma gondii* is distributed worldwide.		
False	29	15.1
True	147	76.6
I do not know.	16	8.3
*Toxoplasma gondii* infects a wide range of animals and birds.		
False	82	42.7
True	86	44.8
I do not know.	24	12.5
Which animals are definitive hosts of *Toxoplasma gondii?*		
Birds	19	9.9
Felids	161	83.8
Reptiles	4	2.1
Rodents	7	3.6
I do not know.	13	6.8
Which of the following clinical manifestations correspond to toxoplasmosis.		
Lymphadenopathy	55	28.6
Ocular disease	52	27.1
Encephalitis	85	44.3
I do not know.	39	20.3

**Table 3:** Knowledge of diagnosis and treatment of toxoplasmosis.

Questions and Answers	No.	%
Which of the following interpretations about the laboratory diagnosis of infection with *Toxoplasma gondii* are correct?		
The demonstration of anti-*Toxoplasma* IgG and IgM in a pregnant woman means invariably that infection was acquired during pregnancy.	69	35.9
A positive test for IgG with a negative test for IgM anti-*Toxoplasma* means a chronic infection.	63	32.8
A negative test for IgG with a positive test for IgM anti-*Toxoplasma* is conclusive of acute infection.	53	27.6
I do not know.	45	23.4
The routine test for detection of anti-*Toxoplasma* IgM antibodies in serum yields a high number of false positive results.		
False	50	26.0
True	62	32.3
I do not know.	80	41.7
Do you know what the anti-*Toxoplasma* IgG avidity test is?		
Yes	19	9.9
No	173	90.1
What is the anti-*Toxoplasma* IgG avidity test used for?		
Good answer	5	2.6
Wrong answer	13	6.8
I do not know.	174	90.6
Do you know another laboratory test (not mentioned earlier) for the diagnosis of infection with *Toxoplasma gondii*?		
Yes	31	16.1
No	161	83.9
If yes, which tests?		
No answer	7	22.6
Good answer	14	45.2
Wrong answer	10	32.2
The demonstration of anti-*Toxoplasma* IgG and IgM antibodies in the serum of a pregnant woman is enough reason to provide treatment.		
False	36	18.8
True	107	55.7
I do not know.	49	25.5
The demonstration of anti-*Toxoplasma* IgM antibodies without IgG in the serum of a pregnant woman is enough reason to provide treatment.		
False	56	29.2
True	81	42.2
I do not know.	55	28.6

**Table 4:** Knowledge of the epidemiology of toxoplasmosis.

Questions and Answers	No.	%
Infection with *Toxoplasma gondii* can be acquired by?		
Ingestion of water	25	13.0
Ingestion of raw or undercooked meat	28	14.6
Ingestion of unwashed fruits or vegetables	26	13.5
Blood transfusion and organ transplantation	52	27.1
Contact with cats	96	50
I do not know.	9	4.7
It is estimated that the world prevalence of *Toxoplasma gondii* infection is:		
Up to 10% of the population	47	24.5
Up to one-third of the population	38	19.8
Up to one-half of the population	7	3.6
Up to 75% of the population	9	4.7
I do not know.	91	47.4

**Table 5:** Practices about toxoplasmosis at the laboratory where participants work.

Questions and Answers	No.	%
In the laboratory where you work, are tests for detection of infection with *Toxoplasma gondii* performed?		
Yes	30	15.6
No	162	84.4
Which tests are used in your laboratory for detection of infection with *Toxoplasma gondii* in pregnant women?		
None	55	28.6
Anti-*Toxoplasma* IgG only	17	8.9
Anti-*Toxoplasma* IgM only	8	4.2
Anti-*Toxoplasma* IgG and IgM	110	57.3
Other	2	1
If other, which one?		
No answer	1	0.5
Good answer	0	0
Wrong answer	1	0.5
Is any confirmatory test for *Toxoplasma gondii* infection performed?		
Yes	14	7.3
No	178	92.7
If yes, which one?		
No answer	9	64.3
Good answer	4	28.6
Wrong answer	1	7.1
Are any molecular tests for *Toxoplasma gondii* infection performed?		
Yes	16	8.3
No	176	91.7
If yes, which one?		
No answer	7	43.7
Good answer	9	56.3
Wrong answer	0	0

## References

[1] Saadatnia G., Golkar M. (2012). A review on human toxoplasmosis. Scand. J. Infect. Dis..

[2] Wyrosdick H.M., Schaefer J.J. (2015). *Toxoplasma gondii*: History and diagnostic test development. Anim. Health Res. Rev..

[3] Lüder C.G.K., Rahman T. (2017). Impact of the host on *Toxoplasma* stage differentiation. Microb. Cell.

[4] Hill D., Dubey J.P. (2002). *Toxoplasma gondii*: Transmission, diagnosis and prevention. Clin. Microbiol. Infect..

[5] Montoya J.G., Liesenfeld O. (2004). Toxoplasmosis. Lancet.

[6] Tidy A., Fangueiro S., Dubey J.P., Cardoso L., Lopes A.P. (2017). Seroepidemiology and risk assessment of *Toxoplasma gondii* infection in captive wild birds and mammals in two zoos in the North of Portugal. Vet. Parasitol..

[7] Cenci-Goga B.T., Rossitto P.V., Sechi P., McCrindle C.M., Cullor J.S. (2011). *Toxoplasma* in animals, food, and humans: An old parasite of new concern. Foodborne Pathog. Dis..

[8] Singh S. (2016). Congenital toxoplasmosis: Clinical features, outcomes, treatment, and prevention. Trop. Parasitol..

[9] Figueroa Damián R. (1998). Risk of transmission of infectious diseases by transfusion. Ginecol. Obstet. Mex..

[10] Barsoum R.S. (2006). Parasitic infections in transplant recipients. Nat. Clin. Pract. Nephrol..

[11] Miller C.M., Boulter N.R., Ikin R.J., Smith N.C. (2009). The immunobiology of the innate response to *Toxoplasma gondii*. Int. J. Parasitol..

[12] Pleyer U., Torun N., Liesenfeld O. (2007). Ocular toxoplasmosis. Ophthalmologe.

[13] Gao X.J., Zhao Z.J., He Z.H., Wang T., Yang T.B., Chen X.G., Shen J.L., Wang Y., Lv F.L., Hide G. (2012). *Toxoplasma gondii* infection in pregnant women in China. Parasitology.

[14] Dhombres F., Friszer S., Maurice P., Gonzales M., Kieffer F., Garel C., Jouannic J.M. (2017). Prognosis of Fetal Parenchymal Cerebral Lesions without Ventriculomegaly in Congenital Toxoplasmosis Infection. Fetal Diagn. Ther..

[15] Zhang K., Lin G., Han Y., Li J. (2016). Serological diagnosis of toxoplasmosis and standardization. Clin. Chim. Acta.

[16] Murata F.H.A., Ferreira M.N., Pereira-Chioccola V.L., Spegiorin L.C.J.F., Meira-Strejevitch C.D.S., Gava R., Silveira-Carvalho A.P., de Mattos L.C., Brandão de Mattos C.C., FAMERP Toxoplasma Research Group (2017). Evaluation of serological and molecular tests used to identify *Toxoplasma gondii* infection in pregnant women attended in a public health service in São Paulo state, Brazil. Diagn. Microbiol. Infect. Dis..

[17] Liesenfeld O., Press C., Montoya J.G., Gill R., Isaac-Renton J.L., Hedman K., Remington J.S. (1997). False-positive results in immunoglobulin M (IgM) *Toxoplasma* antibody tests and importance of confirmatory testing: The Platelia Toxo IgM test. J. Clin. Microbiol..

[18] Dhakal R., Gajurel K., Pomares C., Talucod J., Press C.J., Montoya J.G. (2015). Significance of a Positive *Toxoplasma* Immunoglobulin M Test Result in the United States. J. Clin. Microbiol..

[19] Alvarado-Esquivel C., Sethi S., Janitschke K., Hahn H., Liesenfeld O. (2002). Comparison of two commercially available avidity tests for toxoplasma-specific IgG antibodies. Arch. Med. Res..

[20] Barros G.B., Lemos E.M., E Silva-Dos-Santos P.P., Dietze R., Zandonade E., Mineo J.R., de Oliveira Silva D.A., Pajuaba A.C.M., de Souza Gomes M., do Amaral L.R. (2017). Proposed panel of diagnostic tools for accurate temporal classification of symptomatic *T. gondii* infection. J. Immunol. Methods.

[21] Sroka J., Wójcik-Fatla A., Zając V., Sawczyn A., Cisak E., Karamon J., Dutkiewicz J., Bojar I. (2016). Comparison of the efficiency of two commercial kits—ELFA and Western blot in estimating the phase of *Toxoplasma gondii* infection in pregnant women. Ann. Agric. Environ. Med..

[22] Naghili B., Abbasalizadeh S., Tabrizi S., Rajaii M., Akramiyan M., Alikhah H., Naghavi-Behzad M., Piri R., Karkon-Shayan F., Tehrani-Ghadim S. (2017). Comparison of IIF, ELISA and IgG avidity tests for the detection of anti-*Toxoplasma* antibodies in single serum sample from pregnant women. Infez. Med..

[23] Khammari I., Saghrouni F., Lakhal S., Bouratbine A., Ben Said M., Boukadida J. (2014). A new IgG immunoblot kit for diagnosis of toxoplasmosis in pregnant women. Korean J. Parasitol..

[24] Liu Q., Wang Z.D., Huang S.Y., Zhu X.Q. (2015). Diagnosis of toxoplasmosis and typing of *Toxoplasma gondii*. Parasit. Vectors.

[25] Alvarado-Esquivel C., Sifuentes-Álvarez A., Estrada-Martínez S., Rojas-Rivera A. (2011). Knowledge and practices on toxoplasmosis in physicians attending pregnant women in Durango, Mexico. Gac. Med. Mex..

[26] Davis S.M., Anderson B.L., Schulkin J., Jones K., Vanden Eng J., Jones J.L. (2015). Survey of obstetrician-gynecologists in the United States about toxoplasmosis: 2012 Update. Arch. Gynecol. Obstet..

[27] Alvarado-Esquivel C., Campillo-Ruiz F., Liesenfeld O. (2013). Seroepidemiology of infection with *Toxoplasma gondii* in migrant agricultural workers living in poverty in Durango, Mexico. Parasit. Vectors.

[28] Alvarado-Esquivel C., Liesenfeld O., Márquez-Conde J.A., Estrada-Martínez S., Dubey J.P. (2010). Seroepidemiology of infection with *Toxoplasma gondii* in workers occupationally exposed to water, sewage, and soil in Durango, Mexico. J. Parasitol..

[29] Alvarado-Esquivel C., Rojas-Rivera A., Estrada-Martínez S., Sifuentes-Álvarez A., Liesenfeld O., García-López C.R., Dubey J.P. (2010). Seroepidemiology of *Toxoplasma gondii* infection in a Mennonite community in Durango State, Mexico. J. Parasitol..

[30] Alvarado-Esquivel C., Mercado-Suarez M.F., Rodríguez-Briones A., Fallad-Torres L., Ayala-Ayala J.O., Nevarez-Piedra L.J., Duran-Morales E., Estrada-Martínez S., Liesenfeld O., Márquez-Conde J.A. (2007). Seroepidemiology of infection with *Toxoplasma gondii* in healthy blood donors of Durango, Mexico. BMC Infect. Dis..

[31] Alvarado-Esquivel C., Liesenfeld O., Torres-Castorena A., Estrada-Martínez S., Urbina-Alvarez J.D., Ramos-de la Rocha M., Márquez-Conde J.A., Dubey J.P. (2010). Seroepidemiology of *Toxoplasma gondii* infection in patients with vision and hearing impairments, cancer, HIV, or undergoing hemodialysis in Durango, Mexico. J. Parasitol..

[32] Alvarado-Esquivel C., Estrada-Martínez S., García-López C.R., Rojas-Rivera A., Sifuentes-Álvarez A., Liesenfeld O. (2012). Seroepidemiology of *Toxoplasma gondii* infection in Tepehuanos in Durango, Mexico. Vector Borne Zoonotic Dis..

[33] Elmore S.A., Jones J.L., Conrad P.A., Patton S., Lindsay D.S., Dubey J.P. (2010). *Toxoplasma gondii*: Epidemiology, feline clinical aspects, and prevention. Trends Parasitol..

